# Prevention as a Pillar of Communicable Disease Control: Strategies for Equity, Surveillance, and One Health Integration

**DOI:** 10.3390/epidemiologia7010019

**Published:** 2026-02-03

**Authors:** Giovanni Genovese, Caterina Elisabetta Rizzo, Linda Bartucciotto, Serena Maria Calderone, Francesco Loddo, Francesco Leonforte, Antonio Mistretta, Raffaele Squeri, Cristina Genovese

**Affiliations:** 1 Department of Biomedical and Dental Sciences and Morphofunctional Imaging, University of Messina, 98122 Messina, Italy; 2 Department of Prevention, Local Health Authority of Catania, 95027 Catania, Italy; 3 Department of Integrated Hygiene, Organizational, and Service Activities (Structural Department), Health Management, University Hospital Polyclinic “G. Rodolico—San Marco”, 95123 Catania, Italy; 4 Department of Medical and Surgical Sciences and Advanced Technologies “G. F. Ingrassia”, University of Catania, 95124 Catania, Italy

**Keywords:** prevention, global health, policy, communicable diseases, one health, digital health, AMR, vaccine hesitancy

## Abstract

Global health faces unprecedented challenges driven by communicable diseases, which are increasingly amplified by persistent health inequities, the impact of climate change, and the speed of emerging crises. Prevention is not merely a component but the foundational strategy for an effective, sustainable, and fiscally responsible public health response. This paper delves into the pivotal role of core prevention levers: robust vaccination programs, stringent hygiene standards, advanced epidemiological surveillance, and targeted health education. We detail how contemporary technological advancements, including Artificial Intelligence (AI), big data analytics, and genomics, are fundamentally reshaping infectious disease management, enabling superior predictive capabilities, faster early warning systems, and personalized prevention models. Furthermore, we thoroughly examine the imperative of integrating the One Health approach, which formally recognizes the close, interdependent links between human, animal, and environmental health as critical for combating complex threats like zoonoses and Antimicrobial Resistance (AMR). Despite significant scientific progress, persistent socio-economic disparities, the pervasive influence of health-related misinformation (infodemics), and structural weaknesses in global preparedness underscore the urgent need for decisive international cooperation and equitable financing models. We conclude that only through integrated, multidisciplinary, and resource-equitable strategies can the global community ensure effective prevention, mitigate severe socio-economic disruption, and successfully build resilient healthcare systems capable of withstanding future global health threats.

## 1. Introduction

### Prevention as the Foundational Strategy for Global Health

Global health, defined as the cooperative effort to achieve the highest attainable standard of health for all people worldwide, constitutes the primary goal of modern healthcare policy for fostering prosperous, resilient, and equitable societies. The current milieu is characterized by a confluence of accelerating complex challenges, including the re-emergence of established pathogens, the frequent spillover of novel infectious diseases, widening health inequalities, the destabilizing effects of climate change, and the recurring pattern of health crises. Addressing these threats requires a fundamental shift toward an innovative, integrated, and future-oriented paradigm [1]. Within this complex framework, prevention and active health promotion transition from supplementary measures to essential, cost-effective instruments for dramatically reducing the burden of disease and curbing escalating healthcare expenditure [2]. The most innovative tool for achieving global health is the One Health approach, which promotes a unified, holistic view of health [3]. This concept acknowledges the critical interconnectedness and mutual influence of human, animal, and ecosystem health, exemplified by emerging zoonotic diseases such as Ebola, avian influenza, HIV/AIDS, and the recent COVID-19 pandemic, all of which originated from intricate human–animal-habitat interactions [4]. Fundamentally, global health is not restricted to the biomedical domain but inherently involves complex social, economic, and environmental determinants, touching upon sectors such as education, technology, trade, and land use. For this reason, robust intersectoral cooperation and the development of integrated preventive policies are not optional but essential for achieving efficiency and long-term sustainability [5]. The effective control of communicable diseases—which constitute the primary focus of this paper—demands a globally coordinated and proactive strategy centered on clearly defined prevention pillars.

## 2. Core Prevention Levers for Communicable Diseases

The prevention of infectious diseases fundamentally relies on a hierarchical strategy designed to block the onset and curtail the spread of pathogens across populations by controlling vectors, individuals, and the environment [5].

### 2.1. Vaccination Equity: A Public Health Imperative

Vaccination programs represent one of the crowning scientific achievements in public health, rivaled only by the provision of clean water in their historical impact on reducing mortality and driving population growth [6]. They have been instrumental in significantly decreasing mortality and morbidity from numerous infectious diseases—including measles, diphtheria, tetanus, and pertussis—leading to a marked reduction in infant mortality [7]. High-coverage vaccination is the single most effective tool for protecting the most vulnerable populations through the generation of herd immunity, successfully controlling epidemics and pandemics, as was evidenced during the rapid response to SARS-CoV-2 [8]. Programs have continually evolved, adapting to new challenges with the development of novel vaccines against pathogens like Ebola and SARS-CoV-2 [9]. Despite these monumental successes, the most pressing and unresolved aspect is the lack of universal access to vaccines, which remains severely constrained in low- and middle-income countries (LMICs) where vulnerability to infectious threats is highest [10]. To achieve global equity, strengthened international cooperation in manufacturing and distribution is crucial. Simultaneously, high-income countries face the persistent and growing challenge of vaccine hesitancy, fueled by systematic misinformation and the propagation of false claims that erode public trust in science and public health campaigns [11]. Addressing this dual challenge requires both supply-side equity and demand-side communication efficacy [12].

### 2.2. Digital Early Warning Systems and Predictive Analytics

Effective prevention relies on robust epidemiological surveillance and rapidly scalable early warning systems (EWS) [13,14,15]. Modern surveillance extends beyond traditional active and passive methods, increasingly utilizing integrated digital platforms that harness big data and Artificial Intelligence (AI) to monitor and identify emerging outbreak signals with unprecedented speed and precision [16,17,18]. Using advanced machine learning algorithms and deep analysis of massive, disparate epidemiological datasets, AI and predictive analytics can identify subtle, atypical patterns that may elude human observation, effectively anticipating outbreaks before they escalate into pandemics [19]. The digital revolution has delivered powerful tools for public health: mobile health (mHealth) applications, sophisticated wearable devices, and digital platforms enable granular, real-time monitoring of individual and population health. This facilitates personalized preventive interventions and significantly optimizes resource allocation and response times during health emergencies [20,21]. Given that globalization, international travel, and dense transportation networks accelerate the spread of infectious diseases faster than ever before [22,23,24], international cooperation remains the bedrock for effectively addressing these threats, ensuring the prompt sharing of critical data and resources, as demonstrated during the COVID-19 pandemic [25,26].

### 2.3. Antimicrobial Resistance (AMR) Control via Innovation and Stewardship

The evolution of infectious disease management is severely hampered by the escalating global threat of Antimicrobial Resistance (AMR), necessitating a continuous and radical review of therapeutic and preventative strategies [27,28,29,30]. AMR is a critical public health crisis, driven by the emergence of bacterial strains resistant to traditional antibiotics [31,32]. Addressing AMR is multifactorial and requires a holistic approach:

- Antimicrobial Stewardship: Implementing stringent policies to reduce the inappropriate use of antibiotics in both human medicine and, crucially, in intensive animal farming [33].
- Research and Development: Vigorously funding the development of new classes of antibiotics and exploring innovative alternatives. A promising avenue is bacteriophage therapy, which utilizes specific viruses to target and eliminate harmful bacteria without disturbing the host microbiota [34,35].
- Genomic Surveillance: Leveraging genomics and advanced biotechnology (e.g., CRISPR-Cas) for real-time monitoring of pathogen mutations, which enhances epidemiological surveillance and facilitates the rapid development of personalized, effective antibacterial and antiviral treatments [36,37].

### 2.4. Risk Communication and Infodemic Management

The unchecked spread of misinformation poses a significant, persistent obstacle to the efficacy of public health policies. The COVID-19 pandemic clearly demonstrated how “infodemics” could actively sabotage vaccination efforts and erode public trust in scientific institutions [38,39,40]. Health authorities must proactively counter this by adopting scientific communication that is consistently accurate, clear, transparent, and strictly evidence-based, maximizing reach by utilizing digital channels such as social media, podcasts, and online video platforms [41]. Simultaneously, robust public health education is paramount. Educational campaigns must focus on raising population awareness about essential protective behaviors, including hand hygiene, safe sex practices, and proper management of chronic conditions [42]. School health education, in particular, serves as a fundamental platform for inculcating healthy habits early in life, thereby generating a sustained, positive multiplier effect within the wider society [43].

### 2.5. One Health Integration: Bridging Human, Animal, and Environment

The One Health framework is the essential strategic approach for achieving sustainable global health outcomes [44]. This model formally recognizes the close, interdependent links between the health of humans, animals, and the integrity of the ecosystem. The emergence of zoonotic diseases is intrinsically linked to complex interactions involving biodiversity loss, deforestation, and rapid, unplanned urbanization [45]. One Health integration is thus non-negotiable for effective infectious disease management, encompassing:

- Zoonotic Surveillance: Implementing joint human and animal health surveillance to monitor zoonoses and predict spillover events before they cause pandemics [46].
- Water, Sanitation, and Hygiene (WASH): Significantly improving foundational hygiene and sanitation conditions—including access to clean drinking water, adequate sewage systems, and proper waste disposal—which are critical environmental measures necessary to prevent the spread of diseases like cholera, dysentery, malaria, and viral hepatitis [47,48,49].

### 2.6. Financing, Equity, and Socio-Economic Resilience

Communicable diseases impose a severe and complex socio-economic impact, far beyond the immediate morbidity and mortality statistics. They severely reduce labor productivity, dramatically increase direct healthcare costs, and destabilize national and global economies, particularly affecting vulnerable LMICs [50]. The uncontrolled spread of a disease can trigger a catastrophic economic slowdown, disrupting key sectors globally [51]. The core challenge remains the structural inequality in access to essential health services and novel therapies [52,53,54]. Scarce, poorly distributed healthcare resources often fail to meet population needs, forcing many families to forgo necessary care [55].

Mitigating this requires systemic change:

- Resource and Infrastructure Investment: Substantially improving the equitable distribution of resources and investing in healthcare infrastructures capable of implementing diagnostic tools and distributing vaccines on a large scale [56].
- Global Supply Chain Security: Strengthening international cooperation to establish robust protocols that ensure the continuity of essential goods supplies, especially pharmaceuticals and medical devices, during health emergencies [57].
- Psycho-Social Support: Addressing the profound psycho-social impact (fear, anxiety, depression, stigma) associated with infectious diseases (e.g., HIV/AIDS, tuberculosis) through awareness campaigns and the promotion of social inclusion [58].

## 3. The Role of Advanced Diagnostics and an Operational Model

### 3.1. Advanced Diagnostics: The Time-to-Diagnosis Metric

Early diagnosis is the defining factor for rapid containment of infectious diseases [24]. Rapid diagnostic tests (RDTs)—particularly antigen tests—proved instrumental during the COVID-19 pandemic response. Their key advantage lies in the dramatic reduction in the time-to-diagnosis metric, which is crucial for initiating immediate isolation and contact tracing. For instance, a SARS-CoV-2 RDT provided results in 15–30 min, offering a critical operational gain compared to the benchmark molecular test (RT-PCR), which during periods of high demand often required 24 to 72 h for laboratory processing and result delivery. This difference allowed RDTs to function as an essential tool for effective triage and outbreak management at the community level. Furthermore, investments in universal vaccines against highly variable pathogens like influenza and SARS-CoV-2 are vital for future preparedness [59].

### 3.2. Implementation Framework: Logic Model for Action

To bridge the gap between narrative perspective and operational action, a structured logic model/implementation table is essential. This framework aligns prevention strategies with tangible actions and measurable outcomes, creating an actionable roadmap for policymakers (Table 1).

## 4. Conclusions

### A Unified Call for Global Cooperation

Communicable diseases represent a complex, evolving threat demanding a comprehensive, multi-dimensional approach that seamlessly integrates foundational public health measures, advanced emergency management protocols, accelerated research, and decisive healthcare policies [60]. The current trajectory of global health is being fundamentally altered by disruptive innovations in biotechnology, genomics, and digitalization [61]. To effectively combat future emerging threats, it is imperative to fully implement the One Health strategy, invest heavily in the development and rapid deployment of novel therapies (e.g., monoclonal antibodies, personalized medicine), and standardize next-generation surveillance systems globally [62,63,64,65]. This paper serves as an urgent call to action, urging researchers, clinicians, policymakers, and private sector leaders to collaborate actively in the creation and deployment of successful strategies [66,67,68,69,70,71,72,73]. Ultimately, the success of these strategies will not be measured solely by scientific discovery but by their capacity for equitable and coordinated implementation across the globe, thereby ensuring that all populations—regardless of socio-economic status—have access to effective, sustainable, and timely preventive measures.

## Figures and Tables

**Table 1:** Implementation Framework: Linking Levers to Action.

Prevention Lever	Strategic Objective (Outcome)	Key Operational Actions (Input)	Success Indicators (Output Metrics)
Vaccination Equity	Achieve high, sustained, and equitable vaccination coverage globally.	Establish global pooled financing mechanisms (e.g., COVAX 2.0). Implement community-led, trusted health messaging to combat infodemics.	Increase in DTP3/measles coverage in 90% of LMICs. Reduction in measured vaccine hesitancy rates (e.g., SAGE survey).
AMR Control	Decouple infection treatment from the reliance on last-line antibiotics.	Integrate veterinary and human health surveillance of antibiotic usage and resistance patterns (One Health Surveillance). Incentivize private sector R&D for novel antimicrobial classes and alternatives (e.g., phage therapy).	Decrease in the consumption of Highest Priority Critically Important Antimicrobials (HPCIA) in livestock/humans. Increase in the clinical trial pipeline for new-mechanism antibiotics.
Digital Early Warning	Achieve predictive capacity for pandemic threats and reduce alert time.	Adopt AI/Machine Learning models that integrate environmental (e.g., climate, land use) and syndromic data for risk forecasting. Mandate cross-border, real-time data sharing protocols among regional health organizations.	Reduction in the average “time-to-detection” of novel pathogen spillover events (e.g., from 30 days to <7 days). Accuracy score improvement for 6-month seasonal disease forecasts.

## Data Availability

No new data were created or analyzed in this study. Data sharing is not applicable to this article.
